# Three-Dimensional Kinematic Motion of the Craniocervical Junction of Chihuahuas and Labrador Retrievers

**DOI:** 10.3389/fvets.2021.709967

**Published:** 2021-08-20

**Authors:** Lisa Schikowski, Nele Eley, Nicola Kelleners, Martin J. Schmidt, Martin S. Fischer

**Affiliations:** ^1^Department of Veterinary Clinical Sciences, Small Animal Clinic—Surgery, Justus-Liebig-University, Giessen, Germany; ^2^Department of Veterinary Clinical Sciences, Small Animal Clinic—Neurosurgery, Neuroradiology and Clinical Neurology, Justus-Liebig-University, Giessen, Germany; ^3^Institute of Zoology and Evolutionary Research, Friedrich-Schiller-University, Jena, Germany

**Keywords:** dog locomotion, scientific rotoscoping, craniocervical motion, three-dimensional kinematics, cervical spine

## Abstract

All vertebrate species have a distinct morphology and movement pattern, which reflect the adaption of the animal to its habitat. Yet, our knowledge of motion patterns of the craniocervical junction of dogs is very limited. The aim of this prospective study is to perform a detailed analysis and description of three-dimensional craniocervical motion during locomotion in clinically sound Chihuahuas and Labrador retrievers. This study presents the first *in vivo* recorded motions of the craniocervical junction of clinically sound Chihuahuas (*n* = 8) and clinically sound Labrador retrievers (*n* = 3) using biplanar fluoroscopy. Scientific rotoscoping was used to reconstruct three-dimensional kinematics during locomotion. The same basic motion patterns were found in Chihuahuas and Labrador retrievers during walking. Sagittal, lateral, and axial rotation could be observed in both the atlantoaxial and the atlantooccipital joints during head motion and locomotion. Lateral and axial rotation occurred as a coupled motion pattern. The amplitudes of axial and lateral rotation of the total upper cervical motion and the atlantoaxial joint were higher in Labrador retrievers than in Chihuahuas. The range of motion (ROM) maxima were 20°, 26°, and 24° in the sagittal, lateral, and axial planes, respectively, of the atlantoaxial joint. ROM maxima of 30°, 16°, and 18° in the sagittal, lateral, and axial planes, respectively, were found at the atlantooccipital joint. The average absolute sagittal rotation of the atlas was slightly higher in Chihuahuas (between 9.1 ± 6.8° and 18.7 ± 9.9°) as compared with that of Labrador retrievers (between 5.7 ± 4.6° and 14.5 ± 2.6°), which corresponds to the more acute angle of the atlas in Chihuahuas. Individual differences for example, varying in amplitude or time of occurrence are reported.

## Introduction

Each vertebrate species has a distinct morphology and movement pattern, which reflect the adaption of the animal to its habitat (1). Little is known about the actual motion pattern and ranges of the craniocervical junction during natural locomotion in dogs. Scientific insight is largely limited to data derived from cadaver studies on spinal column specimens or studies performed under standard clinical conditions with sedated animals (2–4). Aberrations of the cervical spine, including those of vertebral body morphology, may have an influence on locomotion, movement patterns, and ranges of motion. For this purpose, two different breeds, a toy breed dog and a large breed dog with no predisposition to craniocervical abnormalities, were selected. Scientific insight about movement patterns may have implications for the understanding, diagnosis, and treatment of “craniocervical junction abnormalities,” which encompass several conditions (5–7).

Our research group was able to record preliminary data on the upper cervical spine to begin with four clinically sound Chihuahuas at a walk and a trot (8). Four additional Chihuahuas and a comparison group of three Labrador retrievers allowed us to establish baseline data on *in vivo* three-dimensional (3D) craniocervical motions.

The aim of this prospective study was to perform a detailed analysis and description of 3D craniocervical non-invasive *in vivo* motion analysis during locomotion in clinically sound Chihuahuas (Ch) and Labrador retrievers (L). Data analysis was focused on gait-cycle-related movements during walking as well as naturally occurring active head and neck motion during locomotion. We were especially interested to evaluate whether body size (Ch vs. L) would have any impact on the timing or range of motion (ROM) of stride-cycle-dependent motions of the cranial cervical spine.

## Materials and Methods

### Animals

A total of 15 Chihuahuas and 14 Labrador retrievers were examined first clinically and later in locomotion on a treadmill. The exclusion criteria were abnormal findings on clinical, orthopedic, or neurological examination or dogs with insufficient habituation on treadmill or movements. Eight Chihuahuas with an average age of 38.5 ± 16.3 months were included. The subjects had an average body weight of 2.8 ± 0.6 kg and a withers size of 19.9 ± 2.3 cm at the time of the study. The gender distribution was 1:1 (Table 1).

**Table 1:** Details of the study population: Chihuahuas (Ch), Labrador retrievers (L).

**Dog**	**Sex**	**Age (months)**	**Body weight (kg)**	**Height at withers (cm)**
Ch1	Female	59	2.8	21
Ch2	Female	11	3.7	25
Ch3	Male	35	2.1	20
Ch4	Female	46	2.1	20
Ch5	Male	35	2.4	25
Ch6	Female	25	2.5	21
Ch7	Male	33	3.7	26
Ch8	Male	64	3.0	24
L1	Male	33	41.7	58
L2	Female	19	33.0	57
L3	Male	27	38.0	63

Three Labrador retrievers with an average age of 26.3 ± 5.7 months were examined as the reference breed. At the time of the examination, the average body weight was 37.6 ± 3.5 kg, and the withers size was 59.3 ± 2.6 cm. The gender distribution was 2:1 with two males to one female. All dogs were privately owned (Table 1).

This study was conducted with the owners' consent. All experiments were reviewed and approved by the Ethics Commission of the German states of Thuringia and Hesse. The registration number of the application is (TLV Reg. No.: 22-2684-04-02-075/14).

### XROMM/Scientific Rotoscoping

Scientific rotoscoping, a markerless, non-invasive method of the XROMM methodology (X-Ray Reconstruction of Moving Morphology) (9), was used for kinematic analysis. The scientific rotoscoping workflow is composed of a large number of individual work steps, from which the movement data are subsequently generated. For this purpose, a bony marionette with articular joints is constructed on the basis of computed tomography (CT) data. Biplanar fluoroscopy and high-speed cameras are used to record the dog's movements on the treadmill. Essentially, the bone marionette is matched with the bony silhouette of the X-ray videos. This procedure results in a 3D spinal column that virtually reflects the real movement patterns of the bony structures during locomotion and enables 3D movement measurements with high accuracy (see (10) for a more detailed description).

### Study Design

After inconspicuous general clinical as well as orthopedic and neurological examinations, a CT examination was performed under general anesthesia. Only subjects with an unremarkable neurological and orthopedic examinations were approved for the study. CT scans (Brilliance, Philips, Best, Netherlands, 16-slice helical scanner) of the head, complete spine, and pelvis of each dog were obtained. Settings of 120 kV and 200 mA were used for the investigation. In addition, an MRI scan of the spine was conducted to rule out cervical conditions which potentially may cause gait alterations. For study subjects examined before December 2016, MRI (MRI 1.0 Tesla superconducting system Intera Philips, Netherlands) was used. After that date, the MRI (MRI 3.0 Tesla Magnetom Verio Siemens, Germany) was used in combination with the Syn-spine-coil. Sagittal T2-weighted images of the cervical, thoracic and lumbar spine and transversal T2-weighted images at the craniocervical and lumbosacral junction were acquired. No dogs included in the study did show any signs of craniocervical junction disease or degenerative lumbosacral stenosis at the time of the investigation.

**Figure 1:**
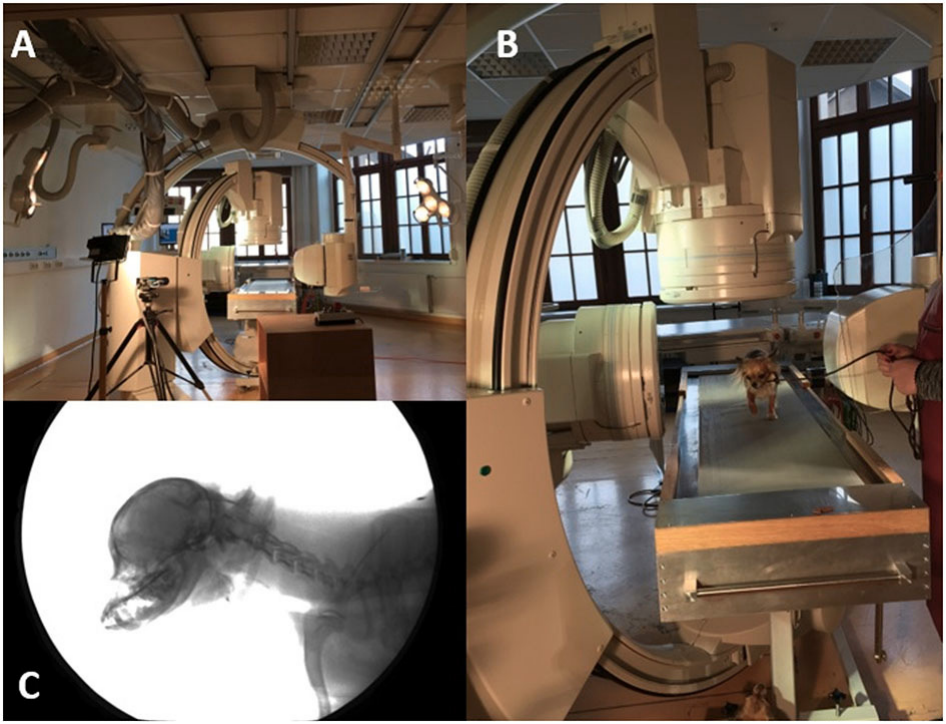
Fluoroscopy 90° experimental setup. **(A)**: 90° fluoroscopy setting to record the-laterolateral and ventrodorsal beam path. The treadmill is located in the center. **(B)**: Chihuahua walking on the treadmill located in the beam path. **(C)**: X-ray video view of the laterolateral beam path.

**Figure 2:**
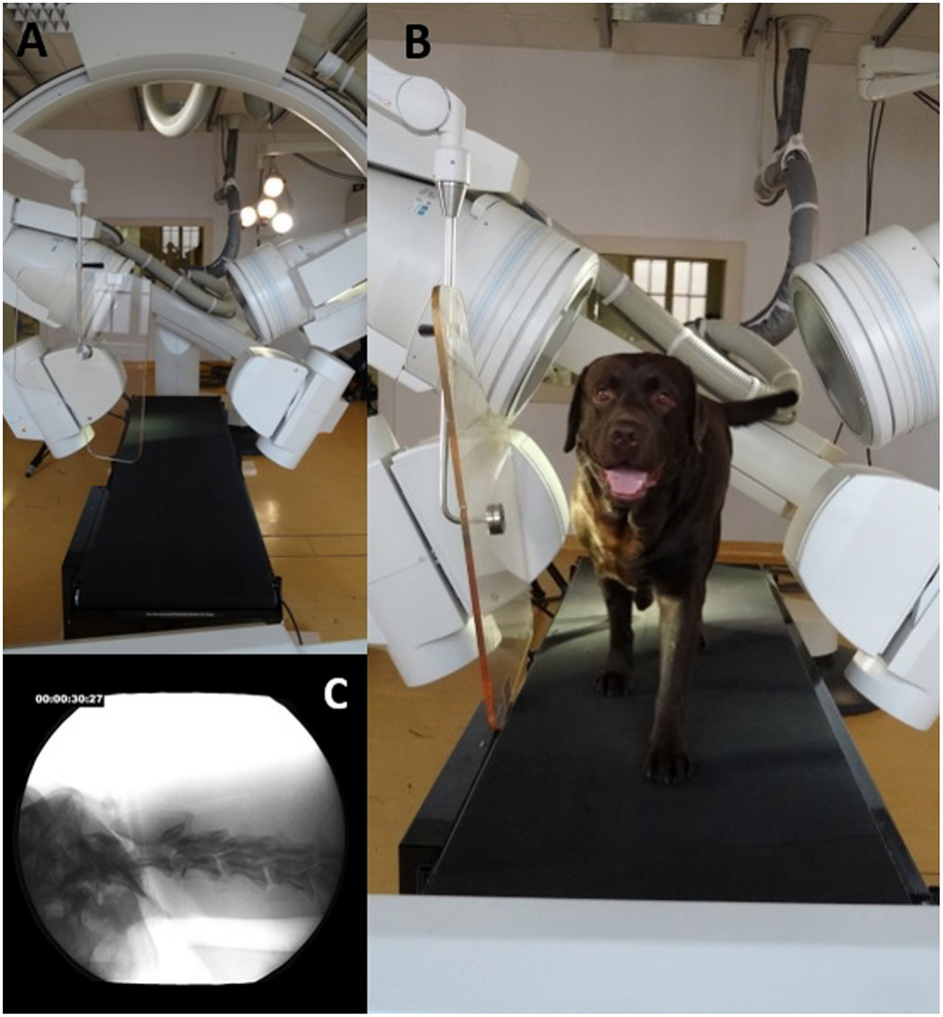
Fluoroscopy 63° experimental setup. **(A)**: 63° fluoroscopy setting to record the oblique-laterolateral beam path. The treadmill is located in the center. **(B)**: Labrador retriever walking on the treadmill located in the beam path. **(C)**: X-ray video view of the oblique-laterolateral beam path.

All subjects were individually habituated to treadmill locomotion. Chihuahuas showed an average walking speed of 0.44 ± 0.09 m/s and Labrador retrievers 0.98 ± 0.2 m/s (Table 2). A horizontal motorized treadmill (Figures 1, 2) was used. The respective duty factor for each dog during walking on the treadmill was calculated (Table 2).

**Table 2:** Overview of Chihuahuas (Ch) and Labrador retrievers (L) regarding the treadmill speed and duty factor in relation to the left hind limb.

**Dog**	**Treadmill speed (m/s)**	**Duty-factor (%)**	**Phase normalization**
Ch1	0.39	66.8 ± 0.8	70/30
Ch2	0.52	63.9 ± 2.0	60/40
Ch3	0.38	62.7 ± 2.2	60/40
Ch4	0.32	63.0 ± 1.3	60/40
Ch5	0.45	65.7 ± 1.5	70/30
Ch6	0.38	67.3 ± 2.7	70/30
Ch7	0.50	58.7 ± 1.1	60/40
Ch8	0.60	62.4 ± 1.3	60/40
**Total Ch**	**0.44 ± 0.09**	**63.8 ± 1.6**	
L1	0.77	62.6 ± 1.7	60/40
L2	0.98	65.3 ± 0.9	70/30
L3	1.20	63.4 ± 0.8	60/40
**Total L**	**0.98 ± 0.2**	**63.8 ± 1.1**	

*Phase normalization of Chihuahuas and Labrador retrievers based on the respective averaged duty factor with respect to the left hind limb*.

Biplanar X-ray videos of each dog during walking were recorded using a digital high-speed videography system. Biplanar fluoroscopy (Neurostar Siemens AG, Munich) consists of two image intensifier systems (diameters 40 cm) as well as high-speed cameras (Visario Speedcam, Weinberger GmbH, Nuremberg). Image resolution is 1,536 × 1,024 pixels at a frame rate of 500 Hz. The C-arms were adjusted in relation to the size of the study subjects, either as a ventrodorsal and a laterolaterale beam (Ch) or in a right and left oblique beam (L) at an angle of 63° (Figures 1, 2) X-ray-settings depended on the dog's size (Chihuahua: 80 kV, 40 mAs; Labrador retriever: 100 kV, 75 mAs). The frame rate was 500 frames per second, and a shutter speed of 500 μs was used to prevent motion blur. The X-ray videos can be viewed in the Supplementary Material.

### Data Analysis

From the recorded trials, consecutive walking strides (*n* = 6) were selected for scientific rotoscoping except for one Labrador retriever (*n* = 3). C3 represents the first animated vertebrae on the hierarchical joint marionette. Because of this hierarchical order C3 reflect the movement of the spine and dog in space and is further called as total upper cervical spine motion (TUCM). The transitional and rotational movements of the TUCM (C3), intervertebral joints (C3/2), the atlantoaxial joint (C2/C1), and the atlantooccipital joint (C1/skull) were analyzed in six degrees of freedom (tx—ty—tz, rx—ry—rz). Due to the individual differences in the duration of the stance and swing phases, a phase normalization (11) with reference to the footfall events had to be performed for all dogs and strides. This allows a comparison of the angular movements across dogs and strides. Time normalization was performed using MATLAB® (TheMathWorks). The duty factor, defined as “the fraction of the duration of a stride for which each foot remains on the ground,” (12) is used for classifying different types of gait. Values >0.5 (contact time >50%) characterize walking (13–15). For each dog and stride, the duty factor was calculated, based on the synchronously recorded high-speed live videos, showing the up and downtimes of the left pelvic limb. The duty factor of Chihuahuas is 63.8 ± 1.6, and that of Labrador retrievers is 63.8 ± 1.1 (Table 2).

The 3D movements of the intervertebral joints and the atlantoaxial and atlantooccipital joint were defined as follows: axial rotations occurred along the horizontal axis, lateral rotations along the vertical axis, and sagittal rotations along the latero-medial axis. The translational movements were only described for the TUCM, which determines the movements in space. Transitional movement could not be detected in the intervertebral joints (C3/2), the atlantoaxial joint (C2/C1), and the atlantooccipital joint (C1/skull). Horizontal translation is translation in the craniocaudal direction; vertical translation, in the dorsal and ventral directions; and lateral translation, in the laterolateral direction.

The arithmetic means and standard deviations of the individual movements, as well as the range of motion (ROM) of each joint, were determined. The movements of the virtual joints of each dog were evaluated and correlated with the stride cycle or other movements that were synchronously observable on the high-speed video. This correlation, the percentage time of occurrence to the stride cycle, is called the time of occurrence (TOO). The movements of the Chihuahuas were compared with each other and with those of the Labrador retrievers. The movements of Labrador retrievers were also correlated.

## Results

### Gait Analysis Using High-Speed Video

Walking speed varied individually between 0.32 m/s and 0.6 m/s for Chihuahuas and between 0.77 m/s and 1.2 m/s for Labrador retrievers. On average, the comfortable treadmill speed of Chihuahuas was 0.44 ± 0.09 m/s, and that of Labrador retrievers was 0.98 ± 0.2 m/s (Table 2). Chihuahuas made an average of 6.19 ± 0.9 strides per minute and Labrador retrievers 4.67 ± 0.3 strides per minute. According to the footfall pattern, the tripod support typically alternated with a parallel or diagonal bipod support during walking.

### Three-Dimensional Movements With Stride Cycle Dependency

**Figure 3:**
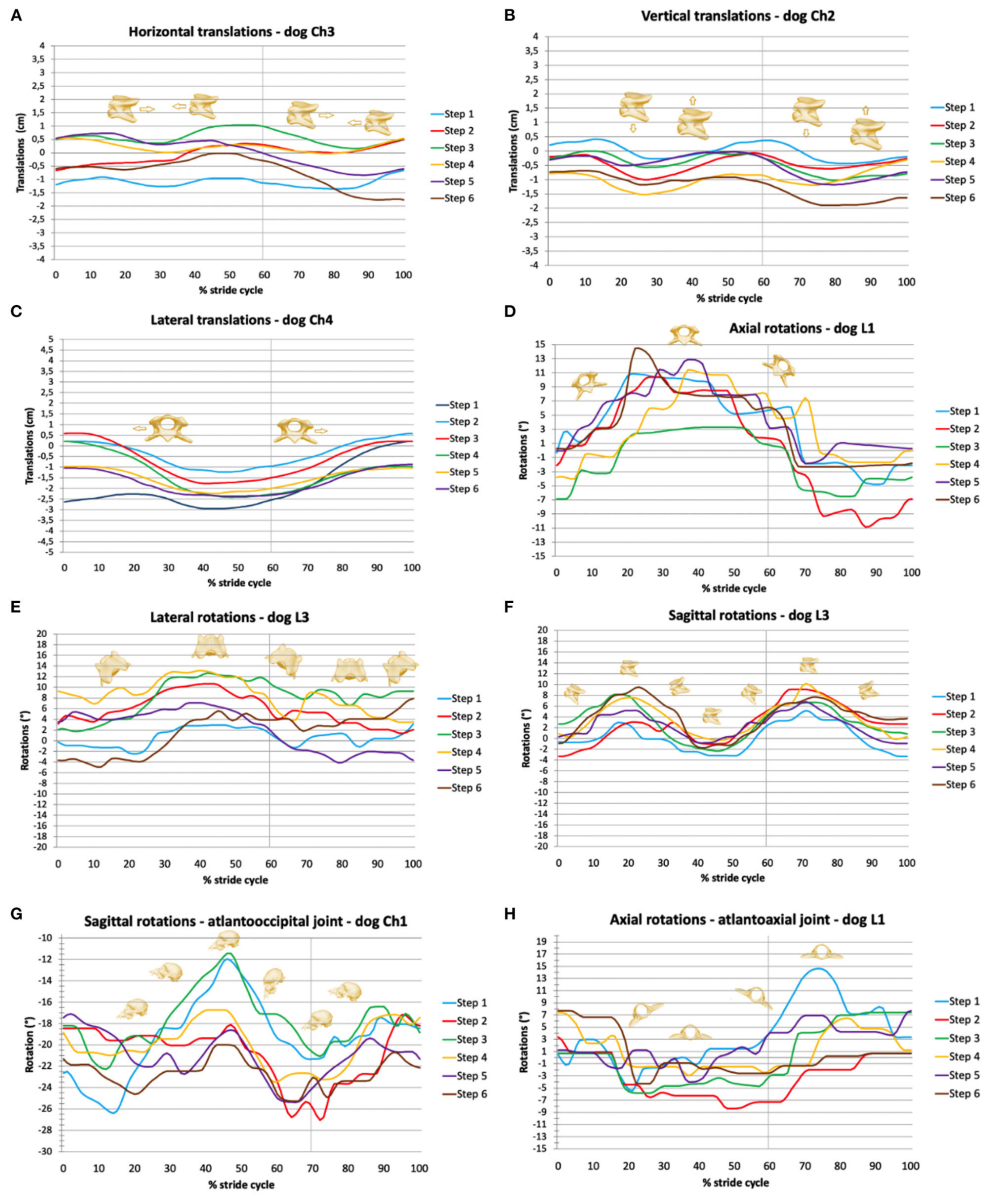
Stride-phase-normalized gait-cycle-dependent movements of the total upper cervical motion (TUCM), atlantoaxial joint, and atlantooccipital joint. Six walking stride cycles (step 1 to step 6) for each dog are presented. X-axes represent the stride cycle from touch-down (0%) to subsequent touch-down of the reference limb (100%). The vertical line indicates the duty factor. **(A)** Horizontal, **(B)** vertical, and **(C)** lateral translations of the TUCM. **(D)** Axial, **(E)** lateral, and **(F)** sagittal rotations of the TUCM. **(G)** Axial rotations of the atlantoaxial joint, **(H)** sagittal rotations of the atlantooccipital joint.

All movements related to the total movements during locomotion were correlated with the stride cycle. Locomotion-dependent movements can be superimposed by active head movements or by position changes on the treadmill. Individual values of TOO and ROM are reported in the Supplementary Tables 3–7. Exemplary stride-cycle-dependent movements are shown in Figure 3.

### Data Analysis of the Total Upper Cervical Motion

**Figure 4:**
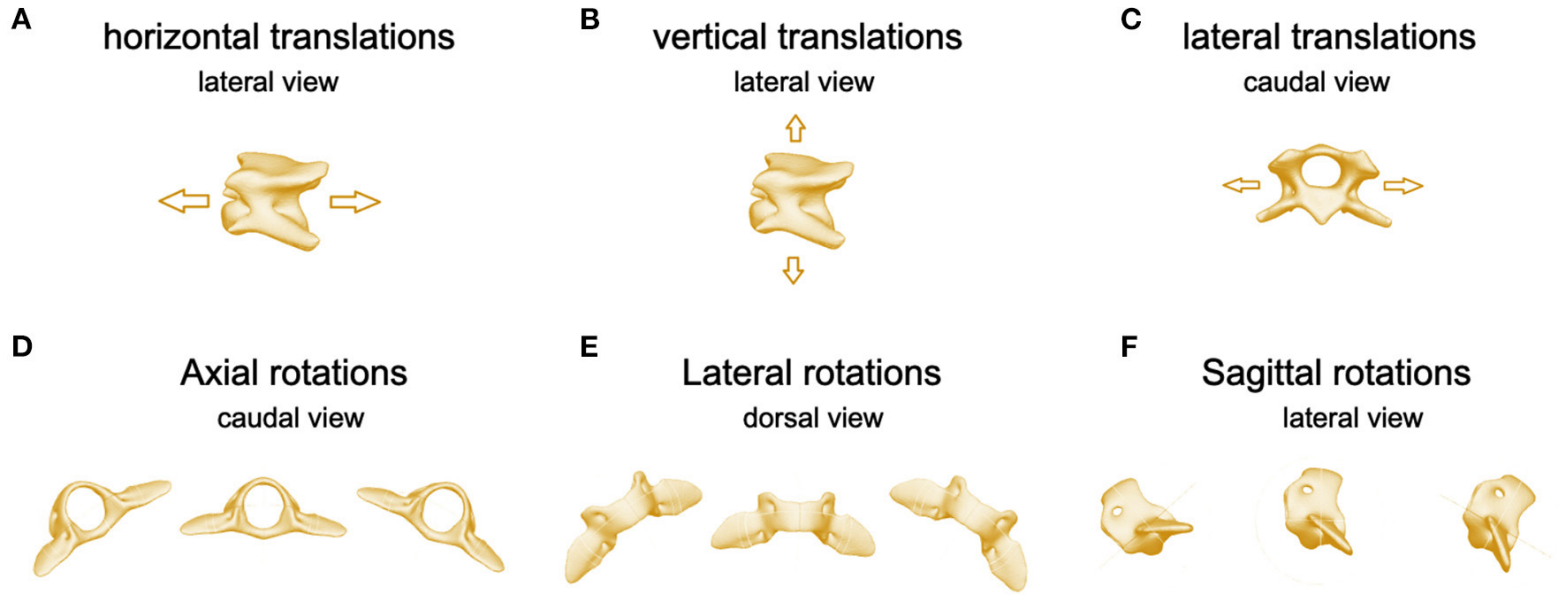
Images of C3 illustrating **(A)** horizontal translations, lateral perspective, **(B)** vertical translations, lateral perspective, **(C)** lateral translations, cranial perspective. Images of C2 illustrating **(D)** axial, **(E)** lateral, and **(F)** sagittal rotations.

Horizontal translation in the craniocaudal direction (Figures 3, 4) showed a biphasic pattern in both breeds. The amplitude and average ROM of Chihuahuas was 0.5 ± 0.7 cm, which was slightly lower than the ROM 0.8 ± 0.9 cm of Labrador retrievers. The highest amplitude was similar in both breeds (5 and 5.2 cm in Chihuahuas and Labrador retrievers, respectively). Horizontal translation in Chihuahuas changed direction on average after 16.3 ± 10.5%, 36.0 ± 6.6%, 60.7 ± 7.3%, and 87.9 ± 6.1% of the stride cycle of the reference limb and in Labrador retrievers after 28.8 ± 0.3%, 40.8 ± 7.5%, 72.1 ± 8.3%, and 91.0 ± 9.9. A change in position of the bony marionette in the cranial direction is associated with lift-off events of the pelvic limbs. A caudal displacement is visible at mid-stance of the pelvic limbs or at the beginning of the second half, at the level of the ipsilateral forelimb lift-off.

A biphasic pattern of vertical translation (Figures 3, 4) was recognized in both breeds. In comparison with horizontal translation, vertical translation showed greater differences with respect to the footfall pattern. The average ROM of Chihuahuas (0.7 ± 0.9 cm) was slightly lower than that of Labrador retrievers (0.9 ± 1.0 cm). The greatest ROM of Chihuahuas (5.8 cm) was approximately the same as that of Labrador retrievers (5.9 cm). Vertical translation in Chihuahuas changed direction on average after 17.6 ± 7.3%, 39.3 ± 10.6%, 67.6 ± 6.5%, and 89.2 ± 6.6% of the stride cycle of the reference limb and in Labrador retrievers after 11.8 ± 8.6%, 36.4 ± 7.9% 60.6 ± 9.3%, and 81.5 ± 9.4%. A vertical translation in the dorsal direction is visible in the swing phase of the anterior limbs. A ventral vertical translation occurs at the beginning of the stance phase of the respective anterior limbs.

A monophasic pattern of lateral translation (Figures 3, 4) was visible in both breeds. The average ROM of Chihuahuas (1.0 ± 1.0 cm) was equal to that of Labrador retrievers (1.0 ± 1.9 cm). The maximum values were significantly smaller for Chihuahuas (4.6 cm) than for Labrador retrievers (12.9 cm). With a weight shift at the beginning of the respective forelimb stance phase, the change in motion direction was associated with right- and left-lateral translation. The direction of translation corresponded to the limb in the stance phase.

Axial rotation is the rotation around the horizontal axis (Figures 3, 4). Multiple movements that cause axial rotation complicate the identification of stride cycle-associated axial rotation. All Labrador retriever showed a correlation to the stride cycle with a monophasic pattern. A correlation of axial rotation to the stride cycle was only visible in four of eight Chihuahuas. The average ROM of the axial rotation of the TUCM was slightly lower in Chihuahuas (2.6 ± 2.9°) than in Labrador retrievers (3.5 ± 3.9°). The maximal values were 16.4 and 18.2 ° in Chihuahuas and Labrador retrievers, respectively. The axial rotation was associated with the forelimb stance phase and reached its maximum in the last third. The change in motion direction was associated with forelimb lift-off. The direction of rotation was related to the corresponding limb in the stance phase. The monophasic pattern was only suggestively visible in one Chihuahua. At the time of lifting-off, the other three dogs with stride cycle dependency show only curve deflections. The entire amplitude of the monophasic pattern of Labrador retrievers was on average at least two to four times larger.

Lateral rotation describes rotation around the vertical axis (Figures 3, 4). Average ROM was 2.3 ± 2.7° for Chihuahuas and 2.7 ± 3.6° for Labrador Retrievers. The greatest amplitude was 17.4° for Chihuahuas and 21.8° for Labrador retrievers. Overall, both breeds showed a monophasic pattern. Among Chihuahuas, this pattern could only be partially observed due to the small entire amplitude. The entire amplitude of the monophasic pattern of Labrador retrievers is greater in comparison to Chihuahuas. Concerning footfall events, greater variation was observed in lateral rotation. Lateral rotation changed direction on average after 53.1 ± 9.4% and 78.0 ± 9.8% of the stride cycle of the reference limb in Chihuahuas and after 42.0 ± 3.1% and 65.6 ± 4.6% in Labrador retrievers. Rotation to the right/left occurred in conjunction with neck movement and weight shift to the right or left during locomotion. The extent of movement and the starting point of the direction of movement varied among dogs but not between the two breeds.

Sagittal rotation describes rotation about the lateral axis (Figures 3, 4). The average ROM of Chihuahuas was 4.0 ± 5.4° and that of Labrador retrievers was 4.8 ± 4.4°. The maximum value was significantly greater in Chihuahuas than in Labrador retrievers (50.9 and 22.0°, respectively). If a stride cycle-associated pattern of the sagittal rotation was observable, it was biphasic. Sagittal rotation changed direction on average after 22.9 ± 6.2%, 41.5 ± 4.0%, 73.6 ± 10.7%, and 90.5 ± 3.6% of the stride cycle of the reference limb in Chihuahuas and after 24.5 ± 7.1%, 48.1 ± 2.8%, 75.7 ± 6.4%, and 93.4 ± 3.7% in Labrador retrievers. Concerning the stride cycle, dorsal sagittal rotation occurs when the neck is raised at the end of the swing phase. Likewise, ventral sagittal rotation is associated with lowering of the neck at the beginning of the forelimb stance phase. Sagittal rotation is related to vertical translation and both occur together.

### Rotational Movements of the Atlantoaxial Joint and Atlantooccipital Joint

The rotational movements in the atlantoaxial joint are predominantly independent of the stride cycle. However, for all Labrador retrievers and one Chihuahua (Ch2) axial rotation of the atlantoaxial joint showed a stride-cycle-associated monophasic pattern. The average ROM of Ch2 was 3.3 ± 2.0°, and that of Labrador retrievers was 3.8 ± 3.6°. The maximum ROM was 24.3° in Chihuahuas and 15.8° in Labrador retrievers. A correlation with the stride cycle existed in the stance phase of the forelimb with a downward/side-to-side movement of the neck/head. The direction of axial rotation was opposite to that of the forelimb in stance. The overall extent of curve deflection varied with respect to the action of the limb and may be primarily punctate or may occur during the course of the stance phase. However, the maximum curve deflection was visible in all dogs in the last third of the stance phase. The amplitude of the stride-cycle dependent pattern of Labrador retrievers was equal to or slightly larger than that of Chihuahuas, but the entire pattern was easier to follow.

The sagittal rotation of the head was linked to the sagittal rotation of the neck but occurred in opposite rotational direction. A relationship between sagittal head rotation to forelimb action during the stride cycle, and sagittal rotation of the TUCM, was likely based on the coupled occurrence. The average ROM of sagittal rotation of the atlantooccipital joint was 3.09 ± 3.5° in Chihuahuas and 3.8 ± 2.9° in Labrador retrievers. The greatest amplitude was 30.2° in Chihuahuas and in 16.8° Labrador retrievers.

### Three-Dimensional Movements During Active Head Movements

When the dog is in motion, sagittal, lateral, and axial rotation can be detected in both the atlantoaxial and the atlantooccipital joint during head movements. Lateral and axial rotation occurs as a coupled motion pattern. In locomotion, mainly lateral movements, as well as extension and flexion movements of the head, become visible. During active head movements in locomotion, sagittal rotation in the atlantoaxial and atlantooccipital joints is related to flexion and extension movements. When the head is moved actively to the left, lateral rotation to the left and axial rotation to the right occurs in the atlantoaxial joint. In the atlantooccipital joint, this head movement results in lateral and axial rotation to the left.

At the atlantoaxial joint, the widest measured ROM were Ch: 20° and L: 9.7° in sagittal rotation, Ch: 26° and L: 7.8° in lateral rotation, and Ch: 24° L: 15.8° in axial rotation. The average ROM of the atlantoaxial joint in all three directions of rotational movement was greater in Chihuahuas than in Labrador retrievers (axial rotation Ch: 4.2 ± 3.7°, L: 3.8 ± 3.6°, lateral rotation: Ch: 2.8 ± 2.8°, L: 2.1 ± 1.7°, Ch: 2.0 ± 2.1°, L: 1.4 ± 1.8°). Axial rotation of the atlantoaxial joint is inferential and, as expected, the rotation with the greatest motion.

In the atlantooccipital joint, the greatest ROM was Ch: 16° and L: 11.7° in lateral rotation, Ch: 18° and L:12.0° in axial rotation, and Ch: 30° and L:16.8° in sagittal rotation. The average ROM of Chihuahuas was 2.7 ± 2.5 ° in axial rotation and 1.8 ± 1.8° in lateral rotation. The average ROM of Labrador retrievers was 2.9 ± 2.3° in axial rotation and 2.4 ± 2.5° in lateral rotation. In conclusion, the sagittal rotation of the atlantooccipital joint is the rotation with the greatest movement during locomotion.

The positioning during CT examination in dorsal recumbency is the reference position and starting position for scientific rotoscoping, set to 0°. Comparing the absolute position of the atlas of Chihuahuas and Labrador retrievers to the reference position Chihuahuas show a slightly higher absolute average value of the sagittal rotation of the atlas (between 9.1 ± 6.8° and 18.7 ± 9.9°) than Labrador retrievers (between 5.7 ± 4.6° and 14.5 ± 2.6°). Regarding the absolute position of the head Chihuahuas showed overall average absolute values between −22.4 ± 16.4° and −42.9 ± 15.1° in sagittal rotation of the head and Labrador retrievers between −33.3 ± 5.5° and −54.1 ± 3.8°.

## Discussion

### Influence of the Treadmill on Gait Pattern

A long discussion on how gait patterns might be influenced by a treadmill [for a review, see Bockstahler, Skalicky [16]] is of no importance in this study as it is not possible to record biplanar fluoroscopy of the cervical spine without using a treadmill. Although the dogs used in our study were well-trained, the dog itself is the greatest factor influencing the variability of the gait pattern (16). The treadmill speed was individually adjusted to the subject to achieve a consistent gait pattern. The selection of a comfortable speed for each individual is very important, as this is the only way to maintain subject compliance, which also leads to a lower variability of steps among them (16). A steady stride was achieved at an average speed of 0.44 ± 0.09 m/s in Chihuahuas and at 0.98 ± 0.2 m/s in Labrador retrievers. The average treadmill speed in Chihuahuas was similar those reported by Kelleners (8) and Fischer, Lilje (13) (0,41 m/s). For Labrador retrievers, the average treadmill speed was in line with the values of the different treadmill speeds (range from 0,77 to 1,22 m/s) used in the literature of Bockstahler, Skalicky (17), Gustås, Pettersson (18), Wachs, Fischer (19), Kopp (20).

### Relationships Between Movements

Biphasic horizontal translation and monophasic lateral translation each show a relationship to position changes during locomotion. The vertical translation, the sagittal and axial rotation of the TUCM as well as the sagittal rotation of C3/C2, the atlantooccipital joint, and the axial rotation of the atlantoaxial joint show a relation to the stance phase and swing phase of the forelimbs. However, the stride-cycle-dependent movements are only visible if the pattern is not “disturbed” by active head movements. Chihuahuas and Labrador retrievers have the same basic pattern of movements with individually varying amplitude and slightly varying TOO.

There is a positive correlation between stride-cycle-dependent horizontal translation and vertical translation as well as between vertical translation and sagittal rotation of the TUCM. Lateral rotation of the TUCM occurs simultaneously with lateral translation in the same direction. At the end of lateral translation, axial rotation occurs in opposite direction. In comparison with Chihuahuas, Labrador retrievers show a significantly greater amplitude of axial rotation of the TUCM. The stride-cycle-associated axial rotation of the TUCM and the axial rotation of the atlantoaxial joint show an opposite direction of rotation. The stride-cycle-associated sagittal rotation of C3/C3 and C3/C2 have the same direction of rotation with an opposite sagittal rotation of the head.

### Three-Dimensional Movements of the Upper Cervical Spine With Stride-Cycle Dependence

Regarding the greater amplitude of the horizontal translation of Labrador retrievers in comparison with that of Chihuahuas, its greater advance due to its larger size or the variations in its position on the treadmill are possible explanations. The amplitude of lateral rotation of the TUCM in the Labrador retriever is significantly greater than that in the Chihuahua. This is consistent with observations by Loscher and Meyer (21) that the amplitude of head-neck movements decreases with relatively short necks.

The axial rotation of the TUCM follows the rolling motion over the trunk in connection with the action of the forelimbs as well as a coupling to lateral and vertical translation during locomotion. In comparison with Chihuahuas, Labrador retrievers show a clearly monophasic axial pattern. This entire amplitude is on average at least two to four times larger. Looking at the gait pattern of Labrador retrievers subjectively, a more ponderous gait with a further out-stepping and reaching of the forelimbs is evident in comparison with that of Chihuahuas. Fischer et al. (13) examined the kinematic parameters of Chihuahuas. In a comparison of both breeds, the forelimbs of Chihuahuas were found to be deflected a very short distance to touch down and lift off at a walk. In stride, the hindlimb deflection to the rear is very small, but the hindlimb is guided far forward for lifting off (13). The rolling motion over the trunk is possibly influenced by limb chiseling out and reaching out. Further chiseling out and reaching out results in greater diagonal motion and, consequently, greater axial rotation. Therefore, because of the greater amplitude, this is easier to identify in Labrador retrievers. Locomotion-associated rotations have already been published for the pelvis and lumbar spine (19, 20, 22, 23).

The magnitude and timing of sagittal rotation, that is, up and down movements of the head and neck during locomotion, are influenced by the neck-trunk ratio, stride frequency, and locomotion speed (21). Additionally, in horses, there is evidence of energetic benefits of the cycle-associated up-and-down movement of the head/neck (21, 24). As the neck oscillates, weight force is transferred to the forelimbs, and energy expenditure is minimized by a phase shift between head-neck oscillation and trunk oscillation (21). Carrier et al. (25), Carrier et al. (26) addressed the EMG pattern of the shoulder girdle muscles and the EMG pattern of the muscles responsible for forelimb protraction and retraction during locomotion in two studies. Both studies were performed on the trotting dog after treadmill habituation. The protractor muscles showed a different pattern of activity, but the main movement of all muscles as a whole was visible toward the end of the stance phase of the forelimb (26). Associating the biphasic pattern of vertical translation and sagittal rotation of the TUCM with the stance phase of the respective forelimb, instead of the swing phase a curve increase is evident from the first third of the forelimb stance phase. This is coincident with lift-off of the contralateral anterior limb. The maximum curve deflection is traceable in the last third of the stance phase. When sagittal rotation and vertical translation are considered together with the activity pattern of the forelimb protractors, dorsal rotation as well as translation of the neck is found to be related to the activity of the forelimb protractors. These findings emphasize the relationship between head and neck motion during locomotion.

The amplitude of the stride-cycle-dependent pattern of axial rotation of the atlantoaxial joint of Labrador retrievers is equally to or slightly larger than that of Chihuahuas, but the overall pattern is easier to follow. Another possible reason is a greater protrusion and extension of the forelimb (13) as well as a larger amplitude of head and neck movement during locomotion (21).

### Comparison of Chihuahuas and Labrador Retrievers Concerning Anomalies of the Craniocervical Junction

The present study involved random head movements with varying degrees of movement between individuals, and no standardized movements were provoked and measured. Therefore, the average values of ROM and ROM_max_ between breeds cannot be used to assume greater mobility within a direction of rotation of a breed. However, in locomotion, the absolute measured values of the atlas and the skull in comparison with the reference position, the CT position, can indicate different angulations of the vertebral bodies between both breeds of dogs. When sagittal rotation of the atlas is examined, Chihuahuas show overall average absolute values between 9.1 ± 6.8 and 18.7 ± 9.9° and Labrador retrievers between 5.7 ± 4.6° and 14.5 ± 2.6°. Thus, the average value of sagittal rotation of the atlas in Chihuahuas was slightly higher than that in Labrador retrievers, which corresponds to a more acute angle of the atlas. However, the high standard deviation of the Chihuahua indicated a large variance. In a comparison of ROM between large and small dogs, similar ROM values would be expected if the vertebral bodies were scaled, but this would not be due to differences in the morphology of the vertebral bodies (27–29). The results are consistent with subjective observations of gait analysis on the treadmill, whereas Chihuahuas, in comparison with Labrador retrievers, showed a more upright neck posture during locomotion. The large inter-individual variance within Chihuahuas may be due to a different neck posture during locomotion. This may also be caused, to a different extent, individually by different degrees of treadmill habituation, but may also be due to a large variance in the morphology of the vertebral bodies and their rotational position relative to each other, which could be a predisposition of individual dogs to pathologies of the craniocervical junction. However, the standard deviation of Labrador retrievers may be lower than that of Chihuahuas only because of the smaller study population. Due to the more acute angle of the atlas of Chihuahuas, an atlantooccipital overlap during locomotion is less likely.

The positioning during CT examination in dorsal recumbency is the reference position and starting position for scientific rotoscoping, set to 0°. When considering the sagittal rotation of the head, Chihuahuas showed overall average absolute values between −22.4 ± 16.4 and −42.9 ± 15.1° and Labrador retrievers between −33.3 ± 5.5 and −54.1 ± 3.8°. Thus, in comparison with Chihuahuas, Labrador retrievers have a more negative sagittal rotational position of the head during locomotion relative to the CT position. The morphology of the head represents a possible cause. The skull of Labrador retrievers is flatter and can therefore be stretched more during positioning for CT, which could explain the more negative sagittal rotational position.

Comparing the absolute position of the atlas of Chihuahuas and Labrador retrievers to the reference position, Chihuahuas show a slightly higher absolute average value of the sagittal rotation of the atlas (between 9.1 ± 6.8 and 18.7 ± 9.9°) than Labrador retrievers (between 5.7 ± 4.6° and 14.5 ± 2.6°). Considering the position of the atlantoaxial and atlantooccipital joint at CT and during surgical treatment in the context of fixation techniques, it is important to recognize that this position does not correspond to the physiological position during locomotion.

### Comparison of the Results at Walk With the Results at Trot, Focusing on Stride-Cycle-Dependent Movements

The stride-cycle-dependent movements at a trot differ from those at a walk, especially in their amplitude. Horizontal and vertical translation of the TUCM in space are larger at trot than at walk (0.7 ± 0.5 cm horizontal translation and 0.8 ± 0.4 cm vertical translation). The larger values of the above mentioned movements can be explained by the oscillating character of the trot (30, 31). Both movements showed a biphasic motion pattern and a correlation with each other during walking and during trotting. Lateral translation of the TUCM is, however, smaller at a trot than at a walk (0.5 ± 0.6 cm), most likely due to the diagonal limb action while trotting, which minimizes this motion pattern. Sagittal rotation of the TUCM is smaller at a trot, too (2.8 ± 2.8°), thus validating the findings that the oscillating character of the trot originates mainly from the trunk while fixing the head (21). Axial rotation of the TUCM has a similar extent and correlation at a walk and at a trot.

## Conclusion

The results of this study confirm the correlation between head and neck movements and locomotion. Chihuahuas and Labrador retrievers have the same basic pattern of movements with individually varying amplitude and slightly varying TOO. For the first time, a similar pattern of these movements is shown in Labrador retrievers and Chihuahuas. General cyclical position changes at a walk as well as movements of the forelimbs affect the head–neck movement in locomotion when the craniocervical junction is not actively moved.

Biphasic horizontal translation and monophasic lateral translation each show a relationship to position changes during locomotion. Vertical translation and sagittal and axial rotation of the TUCM, as well as sagittal rotation of C3/C2 and the atlantooccipital joint and the axial rotation of the atlantoaxial joint, show a relation to the stance and swing phase of the forelimbs. In the atlantoaxial joint, the widest measured ranges of motion are 20° sagittal rotation, 26° lateral rotation, and 24° axial rotation. In the atlantooccipital joint, the widest measured ranges of motion are 16° lateral rotation, 18° axial rotation, and 30° sagittal rotation. Chihuahuas show a slightly higher absolute average value of the sagittal rotation of the atlas (between 9.1 ± 6.8 and 18.7 ± 9.9°) than Labrador retrievers (between 5.7 ± 4.6 and 14.5 ± 2.6°), which corresponds to a more acute angle of the atlas. When moving, the sagittal, lateral, and axial rotation can be detected in both the atlantoaxial and the atlantooccipital joint during head movements. The lateral and axial rotation occurs as a coupled motion pattern. In comparison with Chihuahuas, Labrador retrievers show a greater entire amplitude of axial and lateral rotation of the TUCM as well as of the atlantoaxial joint.

Physiological measured values of the range of motion during locomotion were determined for the sagittal, lateral, and axial rotation in both breeds of dogs. The present study gives insights into the 3D kinematics of the craniocervical joints in locomotion and in active head movements of Chihuahuas and Labrador retrievers. It provides a basis for further comparative studies and contributes to a better understanding of the physiological conditions and variations between the two breeds of dogs studied. The influence of positioning should be included in any sectional imaging and surgical technique.

## Data Availability Statement

The raw data supporting the conclusions of this article will be made available by the authors, without undue reservation.

## Ethics Statement

The animal study was reviewed and approved by the Committee on the Ethics of Animal Experiments of the Justus Liebig University as well as from the Regierungspräsidium Hessen and Thuringia (Permit No.: 22-2684-04-02-075/14). Written informed consent was obtained from the owners for the participation of their animals in this study.

## Author Contributions

LS, NK, NE, MS, and MF conceived the study. LS, NK, and MF conducted the experiments. LS and NK prepared X-ray data and generated the digital bone models from animal data. LS and NK performed the rotoscoping and analyzed the experimental data. LS, NK, and MF drafted the manuscript. All authors contributed to the interpretation of the results and revised the manuscript.

## Conflict of Interest

The authors declare that the research was conducted in the absence of any commercial or financial relationships that could be construed as a potential conflict of interest.

## Publisher's Note

All claims expressed in this article are solely those of the authors and do not necessarily represent those of their affiliated organizations, or those of the publisher, the editors and the reviewers. Any product that may be evaluated in this article, or claim that may be made by its manufacturer, is not guaranteed or endorsed by the publisher.

## Abbreviations

SR: scientific rotoscoping
TUCM: total upper cervical motion
ROM: range of motion
TOO: time of occurrence
Ch: Chihuahua
L: Labrador retriever
C1, first cervical spine: atlas
C2, second cervical spine: axis
C3: third cervical spine
3D: three-dimensional.
