# Supplementary material for: Three-Dimensional Kinematic Motion of the Craniocervical Junction of Chihuahuas and Labrador Retrievers
Source: Front Vet Sci. 2021 Aug 20;8:709967. doi: 10.3389/fvets.2021.709967 (PMC8417724; doi:10.3389/fvets.2021.709967)
Supplement: Supplementary file 1 [file Data_Sheet_1.pdf]

Supplementary Material

1 Supplementary Data

X-ray videos (Video 1 to Video 8)

2 Supplementary Tables

Table 3: Mean ± standard deviations (mean ± SD in%) of the timing of the directional changes (time of occurrence [TOO]) in translations for the total upper cervical motion (TUCM) per dog and averaged across the breeds during walking. Minimum (min) and maximum (max). Horizontal and vertical translations were primarily biphasic with four changes in motion direction per stride cycle. Lateral translations were primarily monophasic with two changes in motion direction per stride cycle. In blank fields, there was no uniform time detectable. Chihuahuas (Ch) and Labrador retrievers (L).

Time of occurrence of the TUCM							
**Dog**		**horizontal translations**		**vertical translations**		**lateral translations**	
		first turning points	second turning points	first turning points	second turning points	first turning points	second turning points
**Ch1**	**max %**	23.7 ± 2.5	63.0 ± 2.0	19.3 ± 2.5	74.7 ± 1.6	96.8 ± 2.6	
	**min %**	43.7 + 2.8	93.0 ± 4.2	39.2 ± 2.2	91.8 ± 2.7	55.2 ± 7.9	
**Ch2**	**max %**	12.2 ± 3.4	59.6 + 2.6	9.8 ± 3.4	54.3 ± 5.3	83.2 ± 4.2	
	**min %**	40.8 ± 1.8	95.2 ± 3.1	26.5 ± 3.5	79.0 ± 3.6	36.2 ± 5.9	
**Ch3**	**max %**	10.6 ± 4.8	50.3 ± 4.1	23.8 ± 6.4	69.8 ± 6.6	-	
	**min %**	29.5 ± 2.3	84.7 ± 5.8	52.4 ± 10.4	98.6 ± 1.1	43.5 ± 1.9	
**Ch4**	**max %**	8.3 ± 7.2	70.2 + 6.6	18.8 ± 4.6	65.0 ± 1.8	97.8 ± 1.7	
	**min %**	39.4 ± 8.1	79.2 ± 7.7	44.3 ± 6.7	87.3 ± 7.4	44.3 ± 2.8	
**Ch5**	**max %**	-	59.3 + 0.5	17.5 ± 7.1	69.0 ± 6.7	-	
	**min %**	27.7 + 5.6	83.7 + 14.1	39.3 ± 5.1	87.0 ± 4.2	41.5 ± 10.0	
**Ch6**	**max %**	8.6 ± 5.9	71.2 ± 6.9	27.3 ± 4.5	73.8 ± 4.5	3.6 ± 1.1	
	**min %**	33.2 ± 5.4	83.4 + 4.1	54.2 ± 8.0	91.0 ± 7.0	50.2 ± 3.1	
**Ch7**	**max %**	-	58.0 + 8.6	8.0 ± 8.6	70.0 ± 9.1	42.7 ± 9.8	
	**min %**	30.4 ± 6.1	89.0 + 2.7	30.8 ± 7.6	82.8 ± 2.3	91.5 ± 6.2	
**Ch8**	**max %**	34.2 ± 2.7	53.5 ± 3.1	6.7 ± 2.7	64.5 ± 2.7	4.8 ± 3.5	
	**min %**	43.7 ± 1.5	95.2 ± 2.5	27.8 ± 5.1	96.2 ± 1.8	35.8 ± 3.8	
**L1**	**max %**	28.6 ± 3.2	77.0 ± 2.8	10.5 ± 3.5	55.6 ± 4.1	-	
	**min %**	46 ± 2.1	96 ± 4.1	38.5 ± 5.4	80.6 ± 4.8	-	
**L2**	**max %**	8.5 ± 5.1	62.5 ± 6.3	21.0 ± 6.5	71.3 ± 3.2	19.0± 1.4	
	**min %**	32.3 ± 3.7	79.5 ± 7.7	43.0 ± 1.7	91.3 ± 5.5	47.5 ± 0.7	
**L3**	**max %**	29.0 ± 2.6	76.8 ± 3.1	4.0 ± 2.2	54.8 ± 7.2	2.3 ± 1.3	
	**min %**	43.3 ±2.5	97.5 ± 3.6	27.7 ± 4.7	72.6 ± 2.6	69.5 ± 5.4	
**Total Ch**	**max %**	**16.3 ± 10.5**	**60.7 ± 7.3**	**17.6 ± 7.3**	**67.6 ± 6.5**	**-**	**-**
	**min %**	**36.0 ± 6.6**	**87.9 ± 6.1**	**39.3 ± 10.6**	**89.2 ± 6.6**	**35.8 + 7.7**	**-**
**Total L**	**max %**	**28.8 ± 0.3**	**72.1 ± 8.3**	**11.8 ± 8.6**	**60.6 ± 9.3**	**-**	**-**
	**min %**	**40.8 ± 7.5**	**91 ± 9.9**	**36.4 ± 7.9**	**81.5 ± 9.4**	**-**	**-**

Table 4: Mean ± standard deviations (mean ± SD in%) of the timing of the directional changes (time of occurrence (TOO)) in rotations for the total upper cervical motion (TUCM) per dog and averaged across the breeds during walking. Minimum (min) and maximum (max). Sagittal rotations were primarily biphasic with four changes in motion direction per stride cycle. Axial and lateral rotations were primarily monophasic with two changes in motion direction per stride cycle. In axial rotations, Chihuahuas (Ch) with step cycle dependency pattern show only curve deflections (starting point and endpoint is documented). In blank fields, there was no uniform time detectable.

**Time of occurrence of the bone TUCM**							
**Dog**		**axial rotations**		**lateral rotations**		**sagittal rotations**	
		first turning points	second turning points	first turning points	second turning points	first turning points	second turning points
**Ch1**	**max %**	86.6 ± 5.8	23.5 ± 2.9	75.6 ± 2.1		25.6 ± 6.8	83.3 ± 4.6
	**min %**	84.7 ± 2.8	59.3 ± 4.9	4.3 ± 2.8		38.8 ± 6.4	94.6 ± 3.9
**Ch2**	**max %**	23.5 ± 2.9	55.3 ± 4.9	46.8 ± 4.5		15.8 ± 2.9	60.8 ± 5.1
	**min %**	6.3 ± 3.4	83.5	78.0 ± 7.1		39.8 ± 2.7	92.7 ± 6.7
**Ch3**	**max %**	66.2 ± 7.1	83.8 ± 6.5	54.3 ± 5.6		19.8 ± 2.6	88.6 ± 7.8
	**min %**	17.8 ± 2.7		85.3 ± 0.7		47.8 ± 8.1	91.3 ± 9.8
**Ch4**	**max %**	-	-	67.7 ± 8.2		30.6 ± 6.4	63.8 ± 7.1
	**min %**	-	-	89.3 ± 2.2		43.2 ± 6.8	85.4 ± 3.5
**Ch5**	**max %**	43.3 ± 7.8	76.8 ± 4.1	43.2 ± 5.6		-	40.4 ± 3.5
	**min %**	25.7 ± 0.6	85.5 ± 4.4	72.5 ± 4.1		-	-
**Ch6**	**max %**	-	-	-		28.6 ± 4.6	71.5 ± 1.2
	**min %**	-	-	-		56.8 ± 5.9	-
**Ch7**	**max %**	40.4 ± 7.0	71.0 ± 6.5	-		-	-
	**min %**	14.8 ± 3.5	77.2 ± 5.6	-		-	-
**Ch8**	**max %**	34.3 ± 4.6	84.6 ± 3.9	53.7 ± 9.3		17.0 ± 2.9	73.8 + 2.6
	**min %**	15.8 ± 2.7	87.7 ± 3.1	64.8 ± 10.2		38.0 + 4.5	88.5 ± 5.9
**L1**	**max %**	43.0 ± 6.3	-	38.7 ± 5.2		21.2 ± 1.7	73.0 ± 8.6
	**min %**	84.8 ± 4.7	-	81.8 ± 2.4		46.8 ± 4.8	92.0 ± 5.5
**L2**	**max %**	53.0 ± 1.0	-	89.3± 1.5		32.6 ± 2.1	83.0 ± 1.7
	**min %**	92.7 ± 2.1	-	42.0 ± 3.6		51.3 ± 4.0	90.7 ± 1.5
**L3**	**max %**	35.6 ± 7.7	-	42.0 ± 3.1		19.6 ± 2.9	71.0 ± 2.1
	**min %**	62.2 ± 7.7	-	65.6 ± 4.6		46.1 ± 3.3	97.6 ± 1.9
**Total Ch**	**max %**	-	-	**53.1 ± 9.4**		**22.9 ± 6.2**	**73.6 ± 10.7**
	**min %**	-	-	**78.0 ± 9.8**		**41.5 ± 4.0**	**90.5 ± 3.6**
**Total L**	**max %**	**43.8 ± 8.7**	-	-		**24.5 ± 7.1**	**75.7 ± 6.4**
	**min %**	**79.8 ± 15.8**	-	-		**48.1 ± 2.8**	**93.4 ± 3.7**

Table 5: Mean ± standard deviations (mean ± SD in%) of the timing of the directional changes (time of occurrence (TOO)) in rotations for the atlantoaxial joint (C2/C1) and atlantooccipital joint (C1/skull) per dog and averaged across the breeds during walking. Minimum (min) and maximum (max). If a stride-cycle-dependent pattern was visible, sagittal rotations were primarily biphasic with four changes in motion direction per stride cycle. Axial rotations were primarily monophasic with two changes in motion direction per stride cycle. In blank fields, there was no uniform time detectable (no stride cycle dependency). Chihuahuas (Ch) and Labrador retrievers (L).

		**Time of occurrence of the atlantoaxial joint (C2/C1)**		**Time of occurrence of the atlantooccipital joint (C1/ skull)**	
**Dog**		**axial rotations**		**sagittal rotations**	
		first turning points	second turning points	first turning points	second turning points
**Ch1**	**max %**			46.0 ± 1.3	91.8 ± 4.8
	**min %**			14.4 ± 3.2	66.4 ± 2.4
**Ch1**	**max %**			46.0 ± 1.3	91.8 ± 4.8
	**min %**			14.4 ± 3.2	66.4 ± 2.4
**Ch2**	**max %**			37.8 ± 1.6	91.0 ± 1.7
	**min %**			5.0 ± 3.7	57.4 ± 4.9
**Ch2**	**max %**			37.8 ± 1.6	91.0 ± 1.7
	**min %**			5.0 ± 3.7	57.4 ± 4.9
**Ch3**	**max %**				
	**min %**				
**Ch4**	**max %**				
	**min %**				
**Ch5**	**max %**				
	**min %**				
**Ch6**	**max %**				
	**min %**				
**Ch7**	**max %**			37.2 ± 4.8	85.0 ± 6.1
	**min %**				63.4 ± 4.6
**Ch8**	**max %**			42.6 ± 5.2	88.5 + 4.6
	**min %**			15.2 ± 3.1	72.2 ± 3.3
**L1**	**max %**	78.8 ± 10.4		42.7 ± 5.3	90.2 ± 9.4
	**min %**	24.2 ± 2.8		21.6 ± 3.0	57.3 ± 9.1
**L2**	**max %**	89.7 ± 2.1			
	**min %**	40.7 ± 4.9			
**L3**	**max %**	77.6 ± 5.9		33.8 ± 2.5	92.8 ± 5.0
	**min %**	22.5 ± 2.3		20.8 ± 2.6	65.2 ± 5.6
**Total Ch**	**max %**				
	**min %**				
**Total L**	**max %**			**24.5 ± 7.1**	**75.7 ± 6.4**
	**min %**			**48.1 ± 2.8**	**93.4 ± 3.7**

Table 6: Mean ± standard deviations (mean ± SD in cm) of the range of motion (ROM) for the translations of the TUCM during walking (n = 6) per Chihuahua (Ch) and Labrador retriever (L) and averaged across the breeds. Indication of the maximum measured translations per dog in centimeters.

Translations TUCM						
**Dog**	**horizontal translations**		**vertical translations**		**lateral translations**	
	**M ± SD [cm]**	**Max [cm]**	**M ± SD [cm]**	**Max [cm]**	**M ± SD [cm]**	**Max [cm]**
**Ch1**	0.6 ± 0.6	1.9	0.7 ± 0.4	1.5	0.6 ± 0.6	1.9
**Ch2**	0.3 ± 0.2	0.9	0.5 ± 0.3	1.1	0.9 ± 0.8	2.3
**Ch3**	0.6 ± 0.5	1.7	0.6 ± 0.6	2.2	0.9 ± 1.1	3.7
**Ch4**	0.2 ± 0.3	1.1	0.6 ± 0.6	2.4	1.8 ± 0.7	3.2
**Ch5**	0.4 ± 0.7	2.0	0.7 ± 0.8	2.9	1.1 ± 1.2	3.9
**Ch6**	1.2 ± 1.4	5.0	1.3 ± 1.8	5.8	0.9 ± 1.3	4.6
**Ch7**	0.3 ± 0.4	1.1	0.5 ± 0.6	2.3	1.1 ± 1.0	2.8
**Ch8**	0.5 ± 0.5	1.7	0.5 ± 0.3	1.2	1.3 ± 1.0	3.2
**L1**	0.8 ± 0.9	1.9	0.9 ± 1.4	6.0	1.6 ± 3.2	12.7
**L2**	1.2 ± 1.0	0.9	0.8 ± 0.9	2.3	0.8 ± 2.0	4.9
**L3**	0.7 ± 0.7	1.7	0.8 ± 0.5	1.8	0.8 ± 0.9	3.4
**Total Ch**	0.5 ± 0.7	-	0.7 ± 0.8	-	1.0 ± 1.0	-
**Total L**	0.8 ± 0.9	-	0.9 ± 1.0	-	1.0 ± 1.9	-

Table 7: Mean ± standard deviations (mean ± SD in cm) of the range of motion (ROM) for the rotations of the total upper cervical motion (TUCM), the atlantoaxial joint (C2/C1), and the atlantooccipital joint (C1/skull) during walking (n = 6) per Chihuahua (Ch) and Labrador retriever (L) and averaged across the breeds. Indication of the maximum measured rotations per dog in °.

Rotations

	Dog	Bone marionette (C3/C3)		Atlantoaxial joint (C2/C1)		Atlantooccipital joint (C1/skull)	
		**M ± SD [°]**	**Max [°]**	**M ± SD [°]**	**Max [°]**	**M ± SD [°]**	**Max [°]**
**axial rotations**	**Ch1**	2.5 ± 2.1	8.2	3.0 ± 1,9	7.1	1.6 ± 1.7	7.5
	**Ch2**	1.9 ± 2.0	7.0	3.3 ± 2,0	12.1	1.8 ± 1.3	5.3
	**Ch3**	2.2 ± 2.6	12.1	4.8 ± 4,2	14.6	3.3 ± 2.9	11.7
	**Ch4**	2.6 ± 3.1	10.4	4.2 ± 3,08	11.8	3.6 ± 2.6	12.3
	**Ch5**	2.8 ± 2.7	11.8	5.5 ± 4,8	21.9	3.8 ± 3.4	18.0
	**Ch6**	2.6 ± 2.7	16.4	6.0 ± 5,5	24.3	2.5 ± 3.2	17.0
	**Ch7**	3.0 ± 2.7	12.8	3.4 ± 2,7	12.4	2.7 ± 2.1	10.8
	**Ch8**	3.5 ± 3.6	12.2	3.5 ± 3,0	12.4	2.8 ± 1.5	6.2
	**L1**	3.7 ± 4.1	17.8	4.4 ± 3,8	15.8	3.1 ± 2.5	12.0
	**L2**	2.4 ± 2.9	13.0	3.5 ± 4,3	15.2	3.5 ± 2.1	8.3
	**L3**	3.9 ± 4.2	18.2	3.5 ± 3,1	14.6	2.6 ± 2.2	10.0
	**Total Ch**	**2.6 ± 2.8**	-	**4.2 ± 3,7**	-	**2.7 ± 2.5**	-
	**Total L**	**3.5 ± 3.9**	-	**3.8 ± 3,6**	-	**2.9 ± 2.3**	-
**lateral rotations**	**Ch1**	2.1 ± 2.2	11.0	2.4 ± 1,9	6.7	1.7 ± 1.8	10.1
	**Ch2**	1.6 ± 1.4	6.5	2.1 ± 1,4	6.1	1.5 ± 1.3	6.4
	**Ch3**	2.0 ± 2.1	9.8	3.0 ± 2,7	10.3	1.6 ± 0.9	3.9
	**Ch4**	4.2 ± 4.6	17.4	2.5 ± 2,2	11.2	1.5 ± 1.4	6.6
	**Ch5**	2.2 ± 2.3	12.3	3.1 ± 3,1	13.8	2.1 ± 1.8	10.0
	**Ch6**	3.0 ± 3.6	17.0	3.8 ± 4,6	26.0	2.4 ± 2.5	11.8
	**Ch7**	2.1 ± 2.7	13.1	2.4 ± 2,7	11.7	2.5 ± 2.4	16.3
	**Ch8**	2.5 ± 2.2	10.6	2.7 ± 1,9	7.2	1.9 ± 1.5	8.0
	**L1**	2.8 ± 3.5	21.8	2.2 ± 1,8	5.8	2.8 ± 2.9	11.7
	**L2**	4.4 ± 5.7	18.6	2.1 ± 1,7	5.8	2.0 ± 2.1	9.2
	**L3**	2.1 ± 2.5	11.3	2.1 ± 1,6	7.8	2.2 ± 2.0	7.2
	**Total Ch**	**2.3 ± 2.7**	-	**2.8 ± 2,8**	-	**1.8 ± 1.8**	-
	**Total L**	**2.7 ± 3.6**	-	**2.1 ± 1,7**	-	**2.4 ± 2.5**	-
**sagittal rotations**	**Ch1**	2.6 ± 3.1	12.7	1.5 ± 1,5	6.5	2.8 ± 2.7	9.7
	**Ch2**	3.8 ± 3.6	11.5	2.4 ± 1,8	6.1	2.2 ± 1.7	7.9
	**Ch3**	3.1 ± 3.1	13.9	2.3 ± 2,2	7.3	2.6 ± 1.9	8.0
	**Ch4**	2.3 ± 2.1	8.0	2.5 ± 2,1	8.0	2.8 ± 2.7	12.6
	**Ch5**	6.6 ± 13.0	50.9	1.8 ± 1,8	6.9	3.3 ± 46	24.7
	**Ch6**	4.1 ± 5.6	23.9	3.0 ± 3,3	20.0	2.9 ± 2.9	14.7
	**Ch7**	6.3 ± 5.4	17.0	1.6 ± 1,63	5.0	5.1 ± 6.6	30.2
	**Ch8**	5.2 ± 4.3	15.5	1.3 ± 1,3	4.7	3.9 ± 3.3	12.5
	**L1**	5.3 ± 5.5	22.0	1.5 ± 1,5	4.5	4.3 ± 3.2	16.8
	**L2**	2.7 ± 2.7	8.6	2.3 ± 2,7	9.7	3.7 ± 3.1	11.8
	**L3**	5.6 ± 3.6	11.2	1.2 ± 1,4	4.9	4.3 ± 6.8	11.7
	**Total Ch**	**3.9 ± 5.4**	-	**2.0 ± 2,1**	-	**3.1 ± 3.5**	-
	**Total L**	**4.8 ± 4.4**	-	**1.5 ± 1,8**	-	**3.8 ± 2.9**	-
